# Silent steam pop detected by transesophageal echocardiography for premature ventricular contractions originating from the aortomitral continuity

**DOI:** 10.1007/s12574-023-00602-2

**Published:** 2023-04-29

**Authors:** Yuhei Kasai, Jungo Kasai, Takayuki Kitai, Ryo Horita, Junji Morita, Tsutomu Fujita

**Affiliations:** 1 Department of Cardiology, Sapporo Cardiovascular Clinic, Sapporo, Hokkaido Japan; 2 University of Washington, Seattle, WA USA

A 56 year-old man (163.0 cm, 95.0 kg, Body Mass Index: 35.8 kg/m^2^) with dilated cardiomyopathy was admitted for catheter ablation of frequent premature ventricular contractions (PVCs). Catheter ablation was considered necessary due to the patient’s symptomatic PVCs, which were unresponsive to β-blocker medications, and a 24 h Holter ECG showing a high PVC burden of 25%. On the basis of QRS morphology (Fig. 1a), we suspected that the origin of the PVCs was the left-ventricular outflow tract, including the aortomitral continuity (AMC) and the left-ventricular summit. Because the patient had obesity, we performed general anesthesia for airway safety. After intubation, a transesophageal echocardiography (TEE) probe (EPIQ Systems, X7–2 T probe; Philips, Andover, MA) was guided into the mid-esophagus. The three cusps of aortic valve were reconstructed and integrated using intracardiac echocardiography (ICE; SoundStar^™^: Biosense Webster, Diamond Bar, CA). However, intracardiac echocardiography could not visualize the AMC. We applied a SmartTouch Surround Flow catheter (ThermoCool; Biosense Webster) to the AMC. During PVCs, the local bipolar activation potential preceded the QRS onset by 48 ms (Fig. 1b), and a perfect pacemap was obtained. The PVCs disappeared 18 s after starting the first radiofrequency ablation (RFA), which was monitored with TEE. We applied bonus ablation to neighboring points (Fig. 1c). At 55 s after the sixth RFA (30–40 W), TEE detected a silent steam pop without an audible pop (Fig. 1d, Supplemental Video). Therefore, we stopped the ablation. No sudden change in electrical impedance occurred (Fig. 1e). The contact force remained stable during RFA (contact force: 7.8 ± 4.0 g, 35 ± 3.8 W, ablation index: 532 ± 116, initial impedance: 116.2 ± 8.2 Ω, and impedance drop: 11.7 ± 2.3 Ω). TEE showed no pericardial effusion. Figure 1f compares the AMC before and after ablation, suggesting that the AMC was sufficiently ablated (red circle; a bright and thickened myocardium in the ablated area can be found). After confirming that the target PVCs could not be provoked, even by isoproterenol infusion, our session was ended. During a 6-month follow-up, there were no PVCs or late complications, such as pseudoaneurysm and pericardial effusion.

**Fig. 1:**
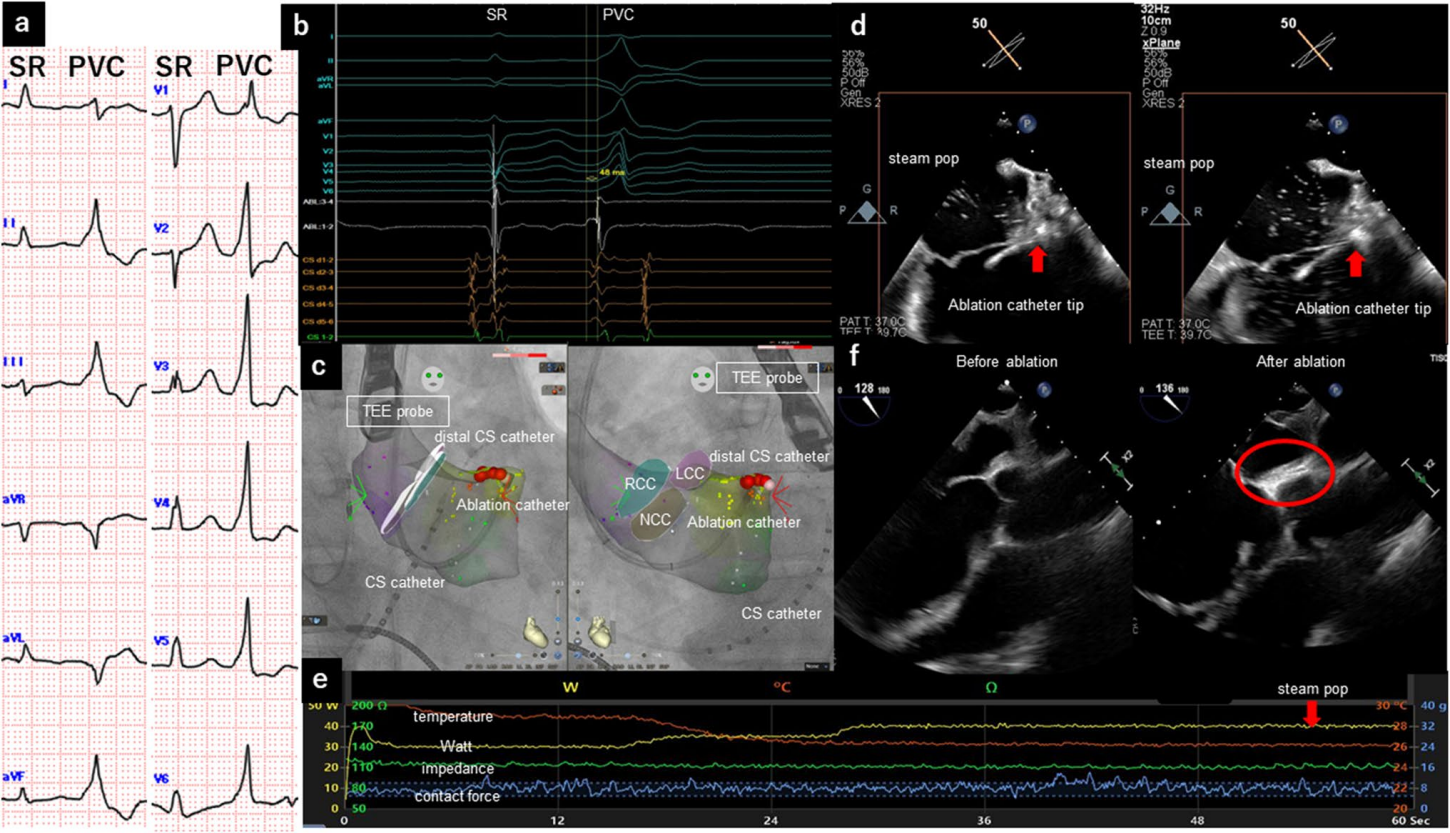
**a** Twelve-lead electrocardiogram comparing the morphology of sinus rhythm and premature ventricular contractions. **b** Intracardiac electrocardiogram, **c** ablation sites and catheter positions, **d** silent steam pop detected by a transesophageal echocardiogram, **e** ablation parameters during the sixth RFA, and **f** transesophageal echocardiogram comparison between before (left) and after (right) ablation

Steam pops are intramural gas bubble explosion caused by overtreatment during ablation. Previous work reports that audible steam pops occur 1.5% of the time during RF catheter ablation, potentially leading to serious complications, such as cardiac tamponade and pseudoaneurysm [1]. Prior work suggests that even when steam pops are inaudible (i.e., silent steam pops), such serious complications can still happen [2]. Moreover, the audibility of steam pops during ablation depends on various factors, such as electrical impedance and electrode temperature [3]. Therefore, if silent steam pops are detected, it is recommended to stop the ablation procedure. Silent steam pops, which can only be diagnosed using echocardiography during RFA, may occur more frequently than originally believed. Intracardiac echocardiography effectively detects silent steam pops [4]. However, the findings in our case suggest that TEE can complement the limitations of intracardiac echocardiography (e.g., AMC in our case) when general anesthesia is applied.

### Supplementary Information

Below is the link to the electronic supplementary material.

Supplementary file1 (MP4 2695 KB)

## Data Availability

The authors confirm that the data supporting the findings of this study are available within the article and its supplementary materials.
